# Superiority of long peripheral catheters over short peripheral catheters for completing planned infusion therapy in late preterm and term neonates: a retrospective cohort study with competing-risk analysis

**DOI:** 10.3389/fped.2026.1808251

**Published:** 2026-04-28

**Authors:** Shigemitsu Kamino, Masafumi Miyata, Chiharuko Nakauchi, Yusuke Funato, Masahiko Manabe, Arisa Kojima, Yuri Kawai, Masayuki Fujino, Hiroko Boda, Tetsushi Yoshikawa

**Affiliations:** 1 Department of Pediatrics, School of Medicine, Fujita Health University, Toyoake, Japan; 2 Department of Neonatology, Tokyo Women’s Medical University Adachi Medical Center, Tokyo, Japan

**Keywords:** catheterization, Infant, Intensive Care Unit, neonatal, peripheral

## Abstract

**Introduction:**

Long peripheral catheters (LPCs) are increasingly used as alternatives to short peripheral catheters (SPCs) in neonatal intensive care units, but their effectiveness for short-term infusion therapy in late preterm and term neonates remains uncertain. This study compared the risk of failure between LPCs and SPCs while accounting for intentional device changes as competing events.

**Methods:**

We conducted a single-center retrospective cohort study between November 2019 and October 2020, including neonates with gestational age ≥34 weeks and birth weight ≥1,500 g who received either a LPC or a SPC as the first venous access device. The primary outcome was infusion failure, defined as premature discontinuation of the index device due to occlusion or extravasation requiring reinsertion or unplanned removal before completion of therapy. Intentional change to another catheter for therapeutic reasons was treated as a competing event. Subdistribution hazard ratios were estimated using Fine–Gray competing-risks regression, and incidence rate ratios per 1,000 device-days were calculated using Poisson regression; cause-specific Cox models were used as complementary analyses.

**Results:**

Of 197 eligible neonates, 66 received LPCs and 131 received SPCs. Median gestational age (37 weeks in both groups) and birth weight (2,750 g vs 2,760 g) were similar. Median dwell time was 3 days in both groups. Excluding intentional changes, failure occurred in 12/58 LPCs (21%) and 48/108 SPCs (44%). The incidence of failure was 62 versus 146 events per 1,000 device-days in the LPC and SPC groups. In the Fine–Gray model, LPC use was associated with a lower subdistribution hazard of failure than SPC use (subdistribution hazard ratios: 0.46, 95% confidence interval: 0.23–0.89). Cause-specific Cox models showed a similar association (adjusted hazard ratio: 0.42, 95% confidence interval: 0.22–0.83).

**Conclusion:**

In late preterm and term neonates requiring short-term peripheral infusion therapy, LPCs were associated with a significantly lower risk of failure and lower failure incidence per device-day than SPCs, even when intentional device changes were considered as competing events. LPCs as a more reliable option for completing short-term planned peripheral infusion therapy in selected neonatal populations, with the caveat that thrombotic complications were not assessed and warrant evaluation in future studies.

## Introduction

Neonatal care in the neonatal intensive care unit (NICU) universally requires reliable vascular access for administration of medications, fluids, and nutritional support (1, 2). Depending on the anticipated duration and characteristics of the infusion therapy, either central or peripheral venous routes may be used. Central venous access, typically via peripherally inserted central catheters (PICCs) or umbilical venous catheters (UVCs), is preferred for long-term therapy or for vesicant and hyperosmolar solutions but carries risks such as infection, thrombosis, and catheter malposition (1, 3). Short peripheral catheters (SPCs) remain the most used devices for peripheral infusion therapy in neonates because they are easy to insert and inexpensive; however, they are prone to early failure due to infiltration, extravasation, and occlusion, often necessitating repeated cannulation and causing tissue injury (4–6). Each re-canulation represents an additional painful procedure for the neonate, contributing to cumulative procedural pain exposure during a period of critical neurodevelopment. In addition, device failures impose a substantial burden on nursing workflow, requiring unscheduled interruptions to care for site assessment, device removal, and reinsertion attempts that may be particularly challenging in neonates with limited peripheral venous access.

Long peripheral catheters (LPCs), which are inserted into deeper peripheral veins with the tip located proximal to the axilla but not in the central circulation, have been proposed as an intermediate option between SPCs and central catheters (7). Several neonatal studies and expert recommendations suggest that LPCs may provide longer indwelling time and fewer device replacements than SPCs, and can help preserve peripheral veins in infants requiring several days of peripherally compatible infusions (1, 4, 7–10). On the other hand, a recent retrospective cohort study by van Rens et al. reported that neonatal LPCs had longer dwell times but higher rates of phlebitis and severe peripheral intravenous infiltration or extravasation compared with SPCs, indicating that potential benefits may be offset by specific local complications and that device performance depends on catheter design, insertion site, and local protocols (11). Thus, the comparative effectiveness of LPCs versus SPCs in neonates remains an area of active debate.

When comparing peripheral devices used for short-term therapy, an important methodological challenge is that catheters can be discontinued for multiple reasons. Some removals are unplanned failures due to device problems, whereas others are intentional changes reflecting planned modifications in therapy, such as escalation to PICC for prolonged treatment or elective removal after completion of the planned infusion course. Previous neonatal studies of midline or long peripheral catheters have generally evaluated catheter dwell time and complication rates without explicitly accounting for these competing events, which may bias estimates of device failure risk. Competing-risks regression, such as the Fine–Gray model, allows estimation of the subdistribution hazard of failure while treating intentional device changes as competing events, providing more clinically relevant estimates of the probability of unplanned failure under real-world NICU conditions (12).

Therefore, we conducted a single-center retrospective cohort study of late preterm and term neonates requiring short-term peripheral infusion therapy to compare LPCs and SPCs with respect to unplanned infusion failure, while treating intentional device changes as competing risks. Our primary objective was to determine whether the choice of peripheral venous access device (LPC vs SPC) was associated with a lower risk of unplanned failure and a higher likelihood of completing planned therapy.

## Methods

### Study design and setting

This was a single-center retrospective cohort study conducted in the neonatal intensive care unit (NICU) of Fujita Health University Hospital between November 1, 2019, and October 31, 2020.

### Participants

We included neonates with a gestational age ≥34 weeks and birth weight ≥1,500 g who required intravenous infusion therapy and received either a LPC or a SPC as the first venous access device after NICU admission. Neonates in whom a PICC or UVC was selected as the initial device, and those admitted for short diagnostic evaluation with a planned infusion duration <24 h, were excluded.

### Device and procedures

At our institution, SPCs were typically selected for short-term peripheral infusion, whereas PICCs or UVCs were chosen when an infusion duration >7 days or the use of vasoactive agents or highly irritant solutions (e.g., parenteral nutrition) was anticipated. LPCs were introduced on April 15, 2020, and thereafter were recommended as an alternative peripheral device when available; however, the final choice between LPC and SPC remained at the discretion of the attending physician. A 24-gauge Surflo® intravenous catheter (Terumo, Tokyo, Japan) was used as the SPC, and an 8-cm Argyle Fukuroi PI Catheter Kit II (Covidien Japan, Tokyo, Japan) was used as the LPC. For the first 48 h of life, a 10% dextrose solution was used as the primary infusion. From postnatal day 3 onward, this was replaced by a maintenance solution containing 10% dextrose with electrolytes.

### Definitions and outcomes

The primary outcome of this study was unplanned infusion failure of the index device (LPC or SPC). “Failure” was defined as premature discontinuation of the index device due to occlusion, infiltration, or extravasation leading to interruption of infusion and requiring reinsertion of a new intravenous route or unplanned device removal before completion of the planned therapy period. Infiltration and extravasation were identified by nursing staff during routine assessment of the insertion site, which was performed at each nursing shift in accordance with institutional NICU protocols. “Success” was defined as continuation of infusion with the index device throughout the planned treatment period. “Intentional change” was defined as elective replacement of the index LPC or SPC with another venous access device (e.g., PICC, UVC, or a new peripheral catheter) based on a planned change in the treatment strategy, such as anticipated prolongation of therapy or need for central administration of irritant drugs, in the absence of device-related failure. Secondary outcomes included dwell time of the index device, reasons for removal, and the incidence rate of failure events per 1,000 device-days. Clinical background variables collected were gestational age of week, birth weight, sex, Apgar score at 5 min, mode of delivery, out-of-hospital birth, postnatal age at initiation of infusion therapy, intravenous access site (dorsal metacarpal veins, cephalic vein of the dorsum of the hand, median antebrachial vein, median cubital vein, lateral marginal vein, medial marginal vein, and others) and primary diagnosis at admission.

### Statistical analysis

Baseline characteristics were compared between the LPC and SPC groups using Fisher's exact test for categorical variables and the Mann–Whitney *U* test for continuous variables. The rate and incidence of failure events per 1,000 device-days were compared using Poisson regression, with log(device-days) as an offset to estimate incidence rate ratios and 95% confidence intervals.

Because intentional device changes may preclude observation of failure, we adopted a competing-risks framework. Time was measured from insertion of the index device to the first occurrence of unplanned failure, intentional change, or censoring. Subdistribution hazard ratios for failure were estimated using the Fine–Gray proportional subdistribution hazards model, treating intentional change as a competing event and adjusting for clinically relevant covariates (gestational age ≥37 weeks, Apgar score ≥8 at 5 min, sex, and mode of delivery). As a complementary analysis, cause-specific Cox proportional hazards models were fitted, censoring at intentional change. Proportional hazards assumptions were examined in analogous Cox models using log–log survival plots and Schoenfeld residual tests. Some evidence of non-proportionality over time was observed for device type; therefore, Cox models were interpreted as complementary, descriptive analyses, whereas Fine–Gray subdistribution hazards were prespecified as the primary analysis. Cumulative incidence functions for failure, with intentional change as the competing event, were plotted for each device group. All analyses were performed using EZR version 1.63; two-sided *p* values <.05 were considered statistically significant.

The primary analysis was the Fine–Gray model of subdistribution hazards; Poisson regression and cause-specific Cox models were prespecified as complementary analyses to assess the robustness of the association between device type and unplanned failure.

### Ethics

This retrospective study was conducted in accordance with the Declaration of Helsinki and was approved by the Ethics Committee of Fujita Health University (approval No. HM23-247). As this was a retrospective observational study using existing clinical records, the ethics committee waived the requirement for individual informed consent. In accordance with Japanese national ethical guidelines for medical research, study information including the research objectives, data items collected, and the right to withdraw data was disclosed on the hospital website, allowing patients’ families to request exclusion of their data at any time.

## Results

**Figure 1:**
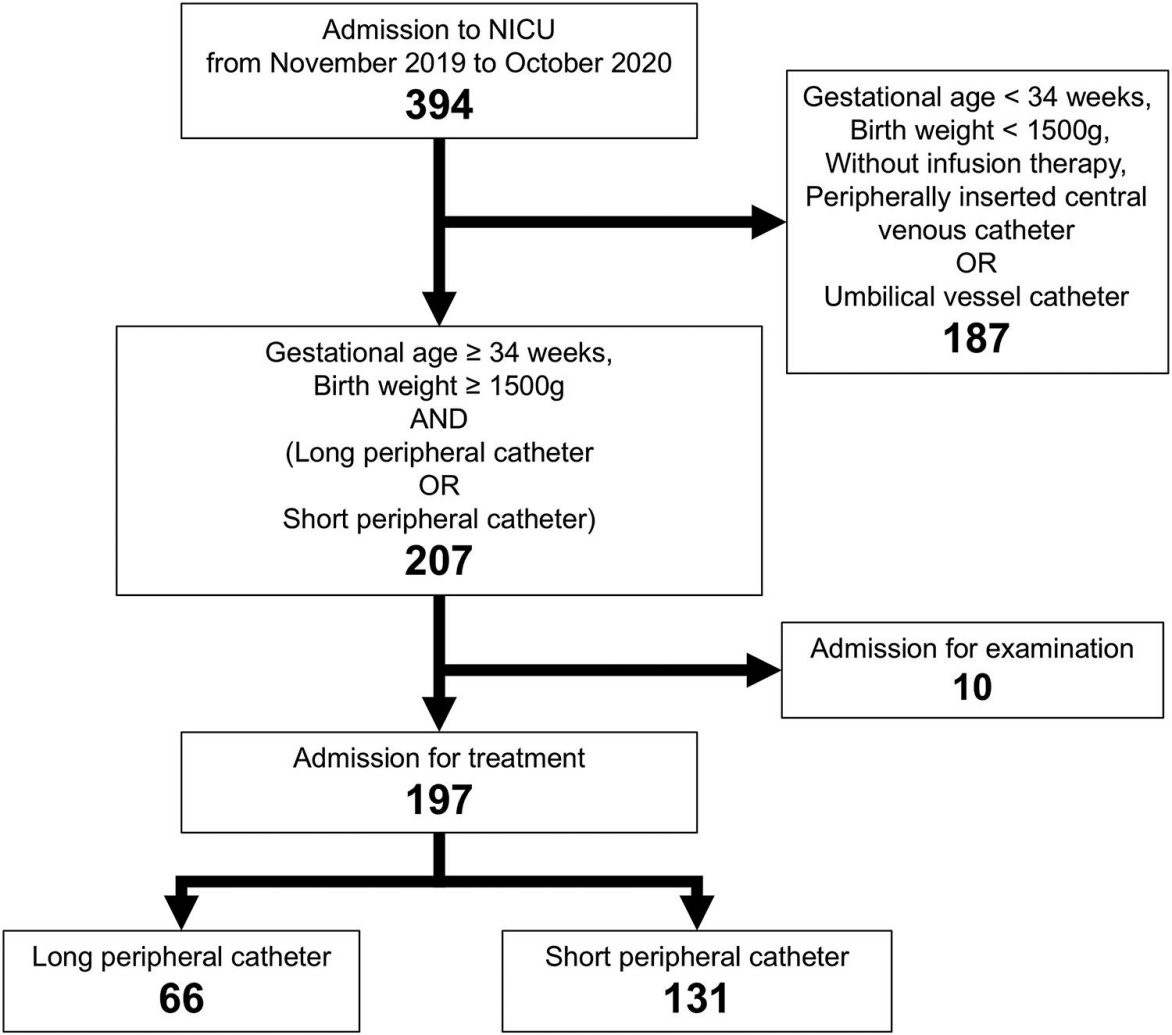
A total of 394 patients were enrolled in the study. Of these, 207 patients were excluded, and 197 patients, including 66 patients in the LPC group and 131 patients in the SPC group, were analyzed. LPC, long peripheral catheter; SPC, short peripheral catheter.

A total of 394 newborns were admitted to the NICU during the study period. Of these, 207 neonates had a gestational age ≥34 weeks, birth weight ≥1,500 g, and received either a LPC or a SPC as the first venous access device. After excluding 10 neonates admitted only for short diagnostic evaluation with a planned infusion duration <24 h, 197 neonates were included in the analysis: 66 received LPCs and 131 received SPCs and (Figure 1). Baseline characteristics, including gestational age, birth weight, sex, Apgar score at 5 min, mode of delivery, out-of-hospital birth, postnatal age at initiation of infusion, and primary diagnosis at admission, were similar between the two groups. Differences were observed between LPC and SPC in the intravenous access site (Table 1).

**Table 1:** Patient characteristics.

	LPC	SPC	*p*-value
	*n* = 66	*n* = 131	
Gestational age (weeks), median (IQR)	37 (36–39)	37 (36–39)	.96
Gestational age ≥37weeks, n (%)	39 (30)	17 (26)	.62
Birth weight (grams), median (IQR)	2,760 (2,334–3,075)	2,750 (2,284–3,806)	.90
Male sex, n (%)	36 (55)	68 (52)	.76
Apgar score at 5 minutes, median (IQR)	9 (9–9)	9 (9–9)	.28
Apgar score ≥at 5 minutes, n (%)	120 (92)	64 (97)	.23
Mode of delivery, cesarean section, n (%)	28 (42)	73 (56)	.10
Out-of-hospital birth, n (%)	29 (44)	69 (53)	.29
Days of age started infusion therapy (IQR)	0 (0–1)	0 (0–2)	.37
Infusion therapy started after 3 days of age, n (%)	8 (13)	33 (22)	.18
Primary admission diagnosis			
Respiratory disorder, n (%)	31 (47)	47 (36)	.17
Feeding disorder, n (%)	8 (12)	27 (21)	.17
Low-birth-weight infant/preterm infant, n (%)	15 (23)	19 (15)	.20
Circulatory disorder, n (%)	1 (2)	7 (5)	.27
Congenital anomaly, n (%)	4 (6)	2 (2)	.10
Others, n (%)	7 (11)	29 (22)	.06
Intravenous access site			
Dorsal metacarpal veins, n (%)	58 (88)	121 (92)	.31
Cephalic vein of the dorsum of the hand, n (%)	0 (0)	1 (1)	1.00
Median antebrachial vein, n (%)	2 (3)	8 (6)	.50
Median cubital vein	2 (3)	0 (0)	0.11
Others, n (%)	4 ()	1 ()	.04

IQR, interquartile range; LPC, long peripheral catheter; SPC, short peripheral catheter.

The median dwell time of the index device was 3 days in both the LPC and SPC groups, with no significant difference between them. When cases with intentional device change were excluded, the proportion of unplanned failure events was significantly lower in the LPC group than in the SPC group (Table 2). The incidence of failure per 1,000 device-days was also lower in the LPC group, and Poisson regression analysis showed a significantly reduced incidence rate ratio for failure associated with LPC use compared with SPC use.

**Table 2:** Details of the infusion status of each device.

	LPC	SPC	p-value	Odds ratio (95% confidence interval)
	*n* = 66	*n* = 131		
Dwell time, days (IQR)	3.00 (2.00–4.00)	3.00 (3.00–3.75)	0.18	—
Intentional change of infusion device, n (%)	8 (12)	23 (18)	.41	.65 (.24–1.62)
Cases excluding “intentional change”	*n* = 58	*n* = 108	p-value	Odds ratio (95% confidence interval)
Failure, n (%)	12 (18)	48 (44)	.002	.33 (0.14–0.72)
Dwell time, days (IQR)	3.00 (2.00–4.00)	3.00 (2.00–4.00)	0.54	—
				Incidence rate ratio (95% confidence interval)
“Failure” event per 1,000 device-days	62	146	.001	0.43 (.32–.57)

IQR, interquartile range; LPC, long peripheral catheter; SPC, short peripheral catheter.

**Figure 2:**
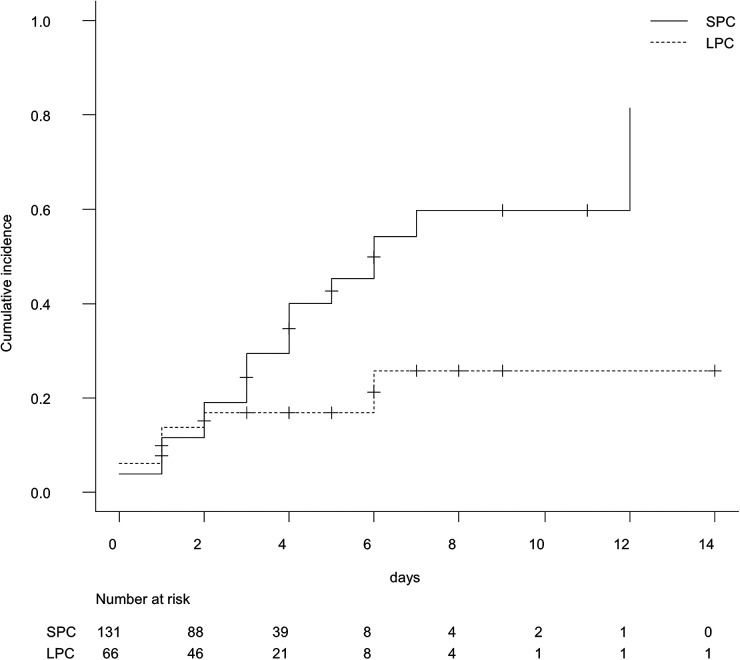
Results of the analysis using the proportional subdistribution hazards model proposed by fine and gray. “Intentional change” was considered a competing risk. The hazard ratio of “Failure” was significantly lower in the LPC group than in the SPC group (subdistribution hazard ratio: .46, 95% confidence interval: .23–.89; *p* = .02). LPC, long peripheral catheter; SPC, short peripheral catheter.

In the Fine–Gray competing-risks model treating intentional change as a competing event, LPC use was associated with a significantly lower subdistribution hazard of unplanned failure than SPC use (subdistribution hazard ratio: .46, 95% confidence interval: .23–.89; *p* = .02). Cumulative incidence function curves demonstrated a consistently lower probability of failure over time in the LPC group compared with the SPC group (Figure 2). No major catheter-related bloodstream infections were identified during the study period; however, the study was not powered to detect rare infection events. In sensitivity analyses using cause-specific Cox proportional hazards models censoring at the time of intentional change, LPC use was also associated with a reduced cause-specific hazard of failure compared with SPC use (adjusted hazard ratio: .42, 95% confidence interval: .22–.83; *p* = .008), although proportional hazards tests suggested some time-varying effects of device type.

## Discussion

In this single-center retrospective cohort of late preterm and term neonates, midline catheters were associated with a lower risk of unplanned infusion failure and a higher likelihood of completing planned short-term peripheral therapy than peripheral intravenous catheters, even when intentional device changes were treated as competing events. The magnitude of the association was consistent across competing-risks regression, Poisson models of failure incidence per device-day, and cause-specific Cox models, suggesting a robust relationship between device type and failure risk.

Previous neonatal studies have reported that LPCs may offer longer dwell times, fewer replacements, and lower rates of extravasation compared with SPCs (1, 4, 8–10), although many did not explicitly account for competing events such as elective removal at therapy completion. More recently, van Rens et al. compared LPC versus SPC in neonates and found that LPC had longer indwelling times but higher rates of phlebitis and peripheral infiltration or extravasation, highlighting that device performance may vary according to catheter design, insertion site, and local protocols (11). Our findings add to this literature by focusing on late preterm and term neonates undergoing short-term peripheral infusion therapy and by using a competing-risks framework to distinguish unplanned failures from planned intentional changes in device strategy.

The competing-risks approach is particularly important in this context because many neonatal patients experience intentional device changes driven by evolving treatment plans, such as escalation to PICC for prolonged therapy or completion of the initial infusion course. Treating these intentional changes as simple censoring can bias estimates of device longevity and failure risk, whereas modeling them as competing events yields more clinically meaningful estimates of the probability of unplanned failure under real-world NICU decision-making. By combining Fine–Gray subdistribution hazards regression with cause-specific Cox models and cumulative incidence functions, this study provides complementary perspectives on how LPCs and SPCs perform when both failure and planned device transitions are considered.

Despite similar median dwell times, the lower failure rate and lower failure incidence per 1,000 device-days in the LPC group suggest that LPCs may provide more reliable peripheral access for completing planned therapy in this selected neonatal population. Each device failure necessitates device removal and reinsertion, exposing the neonate to an additional painful procedural experience. Cumulative procedural pain in the NICU has been associated with adverse neurodevelopmental outcomes, and minimizing unnecessary painful procedures is a recognized priority in neonatal care. In addition, device failures disrupt nursing workflow by requiring unscheduled interruptions for site assessment, device removal, and reinsertion attempts—tasks that are particularly time-consuming in neonates with limited peripheral venous access. Reducing the frequency of such failures may therefore benefit both patients and care teams, contributing to fewer painful cannulations, better preservation of peripheral veins, and a more efficient nursing workflow. At the same time, data from general pediatric populations indicate that thrombotic and mechanical complications are not negligible, with one prospective study observing catheter-related venous thromboembolism in approximately one-third of midlines and mechanical problems in a similar proportion, although most thrombotic events were not severe enough to require systemic anticoagulation and catheter-related bloodstream infection was rare (13). Because our study did not systematically assess catheter-related thrombotic or infectious complications, these safety signals should be interpreted with caution. Nonetheless, our results should not be interpreted as evidence that LPCs are universally safer or superior across all neonatal subgroups, particularly in very preterm or critically ill infants for whom central access is often preferred.

Several limitations warrant consideration. First, the retrospective single-center design and relatively small sample size limit the precision of estimates and the generalizability of the findings. Second, because LPCs were introduced midway through the study period, the study has a non-concurrent cohort structure in which all LPC insertions occurred in the latter half of the observation window while SPC insertions spanned the entire period. This design raises the possibility of temporal confounding from secular trends in clinical practice or operator experience. To assess the impact of this limitation, we performed a sensitivity analysis incorporating calendar period as a time-dependent covariate in the Fine–Gray model. After adjustment for calendar period, LPC use remained associated with a significantly lower subdistribution hazard of unplanned failure compared with SPC use (sHR: .39, 95% CI: .19–.77; *p* = .007), and calendar period itself was not independently associated with failure risk (sHR: .59, 95% CI: .22–1.56; *p* = .29), suggesting that temporal confounding had limited influence on the primary findings. Third, device selection was at the discretion of the attending physician, introducing the possibility of confounding by indication. Clinicians may have preferentially chosen LPCs for patients anticipated to require more reliable or prolonged peripheral access, which could bias results toward LPC superiority. Although measured baseline characteristics were similar between groups, unmeasured factors related to individual clinical judgment—such as perceived difficulty of venous access—cannot be fully excluded as sources of residual confounding. Fourth, infiltration and extravasation were identified through routine nursing assessment rather than a validated structured grading protocol, and no systematic assessment for phlebitis or catheter-related thrombosis was performed. Accordingly, the findings of this study should be interpreted as specific to the outcome of unplanned infusion failure and do not permit conclusions regarding the comparative safety of LPCs and SPCs with respect to thrombotic or inflammatory complications. Fifth, from the third postnatal day onward, a maintenance electrolyte solution with higher osmolarity was commonly used; although the timing of infusion initiation did not differ significantly between groups, osmolarity-related effects on failure risk could not be fully disentangled. Finally, cause-specific Cox models suggested that the relative hazard associated with device type may vary modestly over time, indicating some deviation from the proportional hazards assumption; thus, Cox estimates should be interpreted as complementary to the Fine–Gray subdistribution hazards rather than as definitive time-constant effects.

In conclusion, among late preterm and term neonates requiring short-term peripheral infusion therapy, LPCs were associated with a lower risk of unplanned infusion failure than SPCs when intentional device changes were treated as competing events. These results indicate that, during a short, planned infusion period, LPCs were associated with a lower rate of device failure than SPCs. The present study does not demonstrate that LPCs provide longer dwell times or that they should be maintained for extended periods; rather, the advantage observed here is greater reliability within a short treatment window. These findings support the consideration of LPCs as a more reliable option for completing short-term planned peripheral infusion therapy in carefully selected neonatal patients; however, as thrombotic and inflammatory complications were not systematically assessed, this study does not address the overall safety equivalence of the two devices, while underscoring the need for prospective multicenter studies that incorporate standardized device selection algorithms and systematic monitoring of both mechanical and thrombotic complications.

## Data Availability

The raw data supporting the conclusions of this article will be made available by the authors, without undue reservation.
